# The Research Advances of Aptamers in Hematologic Malignancies

**DOI:** 10.3390/cancers15010300

**Published:** 2023-01-01

**Authors:** Yongkang Liao, Shijun Xiong, Zaid Ur Rehman, Xiaoli He, Hongling Peng, Jing Liu, Shuming Sun

**Affiliations:** Department of Hematology, The Second Xiangya Hospital, Molecular Biology Research Center, Center for Medical Genetics, School of Life Sciences, Hunan Province Key Laboratory of Basic and Applied Hematology, Central South University, Changsha 410011, China

**Keywords:** aptamer, hematologic malignancies, diagnosis, targeted therapy

## Abstract

**Simple Summary:**

Aptamer is a targeting tool with many unique advantages, therefore many experts screened corresponding aptamers in various hematologic malignancies for diagnosis and targeted treatment. In this review, we try to summarize the research progress of aptamers in the diagnosis and treatment of hematologic malignancies to provide support for the application of aptamers in hematologic malignancies.

**Abstract:**

Currently, research for hematological malignancies is very intensive, with many breakthroughs. Among them, aptamer-based targeted therapies could be counted. Aptamer is a targeting tool with many unique advantages (easy synthesis, low toxicity, easy modification, low immunogenicity, nano size, long stability, etc.), therefore many experts screened corresponding aptamers in various hematological malignancies for diagnosis and treatment. In this review, we try to summarize and provide the recent progress of aptamer research in the diagnosis and treatment of hematologic malignancies. Until now, 29 aptamer studies were reported in hematologic malignancies, of which 12 aptamers were tested in vivo and the remaining 17 aptamers were only tested in vitro. In this case, 11 aptamers were combined with chemotherapeutic drugs for the treatment of hematologic malignancies, 4 aptamers were used in combination with nanomaterials for the diagnosis and treatment of hematologic malignancies, and some studies used aptamers for the targeted transportation of siRNA and miRNA for targeted therapeutic effects. Their research provides multiple approaches to achieve more targeted goals. These findings show promising and encouraging future for both hematological malignancies basic and clinical trials research.

## 1. Introduction

Hematological malignancies are heterogeneous tumors affecting the blood, bone marrow, and lymph nodes, including leukemias, lymphomas, myelodysplastic syndromes, and multiple myeloma. They are usually reported to be caused by the uncontrolled proliferation of hematopoietic and lymphoid cells at various stages of their maturation or differentiation and account for 6.5% of all cancers worldwide [1]. Currently, for the treatment of hematologic malignancies, chemotherapy, radiotherapy, and/or immunotherapy regimens have been primarily employed to prevent the tumor from progressing and to prolong the expected patient’s lifespan [2,3,4]. Even though there have been substantial improvements in hematologic malignancies’ five-year survival rates, many challenges remain due to a large number of subtypes and a high degree of heterogeneity [5]. In this case, a potent and specific approach for the treatment of various subtypes of hematologic malignancies would be extremely desirable.

Aptamers are short (15–100 nt) artificial single-stranded oligo(deoxy)nucleotides (ssDNA or ssRNA), which belong to a new class of molecular probes. They have unique and stable three-dimensional structures that allow them to bind specifically to a wide range of targets (e.g., ions, proteins, viruses, and even whole cells and tissues) via three-dimensional binding forces (e.g., hydrogen bonds, aromatic ring stacks, salt bridges, van der Waals forces, and other electrostatic interactions) and shape complementarity to form stable and unique complexes with target [6,7,8,9,10,11,12,13,14,15,16,17]. Aptamers are identified and generated by in vitro molecular methods called SELEX (Systematic Evolution of Ligands by Exponential Enrichment). Aptamers, also known as “chemical antibodies”, offer several exclusive advantages over antibodies (Table 1), such as easier synthesis, low toxicity, easier modification, nanoscale size, low immunogenicity, and a broad range of targets from metal ions to cells, etc. [18,19,20,21,22]. As a novel therapeutic agent, some aptamers bind to target molecules to interfere with signal transduction pathways and regulate cell signaling, thereby inhibiting pathological cell growth or promoting cell death [23]. The other aptamers are commonly used in other biomedical applications such as molecular bioimaging, disease diagnosis, and targeted drug therapies [24].

**Figure 1:**
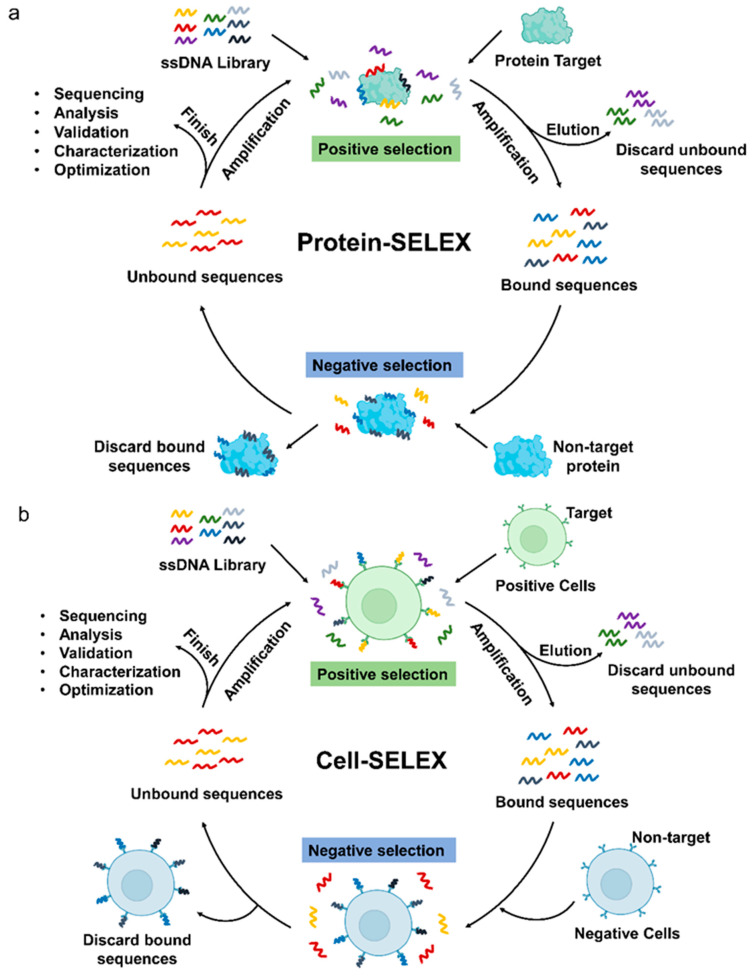
DNA aptamer selection using SELEX. (**a**) Protein-SELEX aptamer selection procedure. Positive selection: Random ssDNA library is incubated with the target protein, then the bound sequences are separated from the unbound sequences, and the bound aptamers are recovered and amplified; Negative selection: Amplified target ssDNA from positive selection is incubated with non-target proteins, bound sequences are discarded, and unbound aptamers are collected and amplified by PCR as an enrichment pool for the next round of selection. The steps are repeated cyclically, followed by sequencing and analysis to isolate high affinity and specificity aptamers. (**b**) Cell-SELEX aptamer selection procedure. Positive selection: Incubate ssDNA library with target cells, discard unbound sequences and amplify bound sequences; Negative selection: The amplified sequences are further incubated with non-target cells, the bound aptamers are discarded and the unbound aptamers are amplified by PCR as an enrichment pool for the next round of selection. After repeating several rounds of selection, the sequences are sequenced, analyzed, characterized, and optimized.

Generally, the discovery and validation of new aptamers rely mainly on SELEX, which allows the identification of aptamers by selection and amplification from a huge pool of nucleic acids composed of randomly assembled libraries [25]. Common SELEX methods fall into two broad categories, Protein-SELEX (Figure 1a) and Cell-SELEX (Figure 1b) [26]. Many specific aptamers have been developed by protein-based SELEX methods. In Protein-SELEX, purified or recombinant proteins are selected as targets for SELEX to achieve optimal enrichment during the selection process. While Cell-SELEX offers a novel option for developing aptamers [27]. Cell-SELEX starts screening with whole cells as targets, and targets on the surface of living cells exist in their natural state during the screening process, which can lead to better recognition or internalization of screened aptamers [28]. In addition, Cell-SELEX allows the discovery of new targets on the cell surface, which is essential for the discovery of small biomarkers for the treatment of diseases. So far, in the aptamer research of hematological malignancies, four aptamers were obtained by Protein-SELEX screening, while Cell-SELEX screening yielded ten aptamers, and three aptamer screens were used in both ways.

Herein, we summarize recent advances in aptamers for the treatment and diagnosis of hematologic malignancies. and then we will discuss their challenges and prospects in clinical diagnostic and therapeutic applications.

## 2. Aptamers in Leukemia

Leukemia is a heterogeneous malignancy of the hematopoietic system characterized by the production of abnormal white blood cells in the bone marrow. Leukemia is mainly divided into two categories, myeloid and lymphoid, depending on the types of affected white blood cells. Four major subtypes are recognized: acute myeloid leukemia (AML), acute lymphoblastic leukemia (ALL), chronic myeloid leukemia (CML), and Chronic Lymphocytic Leukemia (CLL) [26,29]. Next, the research progress of aptamers in each leukemia subtypes from different perspectives will summarized.

### 2.1. Aptamers in AML

AML is an oligoclonal and genetically heterogeneous disease caused by mutations in genes that regulate the ordered proliferation, differentiation, and maturation of hematopoietic cells. It is characterized by impaired bone marrow differentiation and uncontrolled proliferation of immature bone marrow cells, resulting in normal hematopoiesis failure and bone marrow failure [17,30,31,32,33]. Approximately 147,000 deaths due to AML were reported worldwide in 2015, which poses a serious threat to human health [34]. Chemotherapy is the main clinical treatment of AML, but it has side effects such as impairing normal cells and being prone to relapse [35], leading to an overall 5-year-survival rate of only 30–40% [36,37,38]. It is imperative that develops safe and efficient therapy in recent years, novel targeted therapies have been rapid development for AML treatment. There is a growing body of research on AML-targeted therapeutic aptamers. Here, we present a comprehensive overview of the latest advances in aptamers in AML, using different targets as entry points.

#### 2.1.1. Cell Surface Protein Targeted Aptamers

CD123 is a promising biomarker in the AML-targeted drug delivery system as it is expressed at high levels in 45–95% of AML cells and low levels in primitive hematopoietic stem cells, erythroid progenitors, mature granulocytes, and lymphocytes [36,39,40,41,42]. Wu et al. developed two novel CD123 aptamers, ZW25 and CY30, that bind specifically to CD123^+^ AML tumor cells. In addition, the authors constructed a ZW25-mediated targeted drug (TDT) system that can target doxorubicin (Dox) to CD123 cells via the endosomal pathway. Compared with the control group, TDT specifically inhibited CD123^+^ cells in vitro and significantly prolonged the survival of CD123^+^ tumor-bearing mice, inhibited tumor growth, and attenuated the side effects on normal tissues in vivo [43]. Although CD123-aptamer may have excellent affinity and specificity as tumor-binding ligands for AML-targeted therapies, their nuclease digestion limited pharmacological applications. Subsequently, they developed the first CD123 thioaptamer (SS30) to overcome these obstacles [44]. SS30 is the first CY30-derived thioaptamer that binds CD123 with a high specificity while remaining resistance to nuclease digestion. In addition, SS30 selectively inhibited the proliferation of CD123^+^ AML cells in vitro, and reduced the tumor volume of the mouse xenograft tumor model in vivo. In another study by Wu’s team, they constructed an aptamer hydrogel called SSFH that can be precisely cut by Cas9/sgRNA for programmed SS30 release [45]. SSFH-released SS30 inhibits cell proliferation and induces apoptosis in vitro, and inhibits tumor growth via the JAK2/STAT5 signaling pathway in vivo. It is not difficult to find that CD123-aptamer has great potential for application in AML-targeted therapy. Through years of efforts by Wu’s team, many problems of CD123-aptamer in AML targeting therapy have been solved, and aptamer therapy for AML has advanced a great step forward.

CD117, another biomarker for the treatment of AML, is a transmembrane protein receptor expressed at high levels in leukemic cells of almost all patients with relapsed AML [46]. More importantly, among AML patients, CD117^+^ AML patients have a poorer survival prognosis, and high CD117 expression leads to low complete remission rates [47,48,49,50,51]. An ssDNA aptamer was developed for CD117 that not only selectively binds AML cells with high affinity (Kd = 4.24 nM), but also internalizes into cells with high specificity [52]. The aptamer and methotrexate were synthesized into an aptamer-drug conjugate (Apt-MTX) for the targeted therapy of AML. Apt-MTX selectively inhibited AML cell proliferation and induced apoptosis by inducing G1 phase cell cycle arrest compared to the control. Clinical samples have shown that Apt-MTX also specifically inhibits the growth and survival of primary AML cells in patients’ cells.

Oncofetal antigen/immature laminin receptor protein (OFA/iLRP) is a 37 kD membrane protein that is an attractive biomarker for targeted AML therapy [53]. An’s team developed the first OFA/iLRP aptamer (AB3), a 59 nt DNA oligonucleotide that binds to OFA/iLRP-positive AML cells (Kd = 101 nM) and is used to deliver Dox to AML cells [54]. The Apt-Dox compound was created by inserting Dox into the DNA structure of AB3, which effectively inhibited OFA/iLRP positive AML cells compared to control cells.

CD33 is expressed in most adult and pediatric AML progenitor cells as well as adult leukemia stem cells [55]. Yang et al. used positive (CD33-transfected-HEK293T cells) and negative (HEK293T cells) in aptamer selection, which is a modified strategy to the traditional Cell-SELEX [56]. They successfully obtained an aptamer S30 targeting CD33, which showed high recognition of the C2 structural domain of CD33 antigen both in vitro and in vivo. The optimized aptamer S30-T1 was coupled with Dox to form S30-T1-Dox conjugates to inhibit the proliferation of HL-60 cells in vitro by blocking the cell cycle in the G2 phase. Zaimy et al. [57]. evaluated an engineered nanostructure of functionalized gold nanoparticles (FGNs) that contained five antisense oligonucleotides and an anti-CD33(+)/CD34(+) aptamer. This study found that FGNs could treat acute myeloid leukemia subtype 2 (AML-M2) by silencing five essential oncogenes (BAG1, MDM2, Bcl-2, BIRC5, and XIAP) in AML-M2.

#### 2.1.2. Other Protein Targeted Aptamers

All the above mentioned aptamers are cell membrane surface proteins. Aptamers can also select cellular expressed genes as targets. Earnest et al. selected aptamers by SELEX against AML cells expressing the MLL-AF9 oncogene [58]. The authors tested the affinity of the KGE02 aptamer for various AML subtypes to demonstrate that whether the aptamer could be used in clinical samples. They found that five of seven relapsed AML samples displayed high binding affinity, and show no real association between binding and cell karyotype. This suggests that KGE02 can effectively target multiple AML types and shows the prospect of future treatment with clinical drug conjugates.

Some of the aptamers could be used directly as therapeutic agents for diseases. AS1411 is a therapeutic aptamer and is the first ssDNA aptamer to be tested in clinical trials. AS1411 specifically and with high affinity binds nucleolin, which is overexpressed on the membranes of various cancer cells, including AML. AS1411 has been shown to effectively and significantly inhibit the cell growth of the AML cell line MV4-11 and in AML patients [59,60]. Clinical phase II studies (ClinicalTrials.gov: NCT00512083 and NCT01034410) also showed synergistic anticancer effects of AS1411 in combination with cytarabine in AML. In addition, Deng et al. [61] demonstrated that the NCL/miR-221/NF*κ*B/DNMT1 axis is a novel molecular pathway contributing to AML leukemogenesis and prepared a nuclear localization signaling peptide targeted gold nanoparticle co-loaded with anti-221 and AS1411 (NPsN-AS1411/a221). The complex specifically targeted crucial molecules involved in the NCL/miR-221/NF*κ*B/DNMT1 pathway to synergistically inhibit AML cell growth in vitro and in vivo compared to controls.

Among the above aptamer studies in AML, nine aptamers have been used for targeted therapy in AML, mostly by combining aptamers with traditional chemotherapeutic drugs such as DOX, MTX, etc., to achieve targeted therapy by enhancing drug efficacy and alleviating chemotherapeutic side effects. Among all these aptamers, the CD123 aptamer has been deeply studied by Wu’s team, solving many problems that need to be solved for clinical application, while Aptamer AS1411 has shown synergistic effects with cytarabine in AML treatment in clinical phase II. These results fully demonstrate the potential application of aptamers in AML. Most of the existing studies focus on the therapeutic effects of aptamers on AML. Perhaps, researchers can also develop these aptamers into corresponding diagnostic reagents in potential AML patients.

### 2.2. Aptamers in ALL

ALL is the most prevalent type of childhood tumor, representing approximately 80% of total childhood leukemia, with over 349,000 new cases diagnosed worldwide in 2018 [62,63,64]. In addition, the prevalence of ALL is increasing every year and poses a huge threat to human health. The standard regimen for ALL is a combination of chemotherapy, radiation therapy, and surgery. Due to its unsatisfactory curative effect and strong systematic side effects, the current treatment is far from achieving the best effect [65,66]. Nowadays, with the fast development of biomedicine, aptamers hold great promise in treating ALL. Next, the progress of aptamer research in ALL in terms of diagnostic and therapeutic applications will be summarized, respectively.

#### 2.2.1. The Role of Aptamers in ALL Diagnoses

Currently, the improvement of ALL patients’ prognosis is largely dependent on the diagnosis of different malignancy. Therefore, there is a great need to develop a new promising diagnostic technology for ALL. Aptamers have the advantage of being easily modified and truncated, which facilitates the development of cancer diagnostic probes and sensors. A variety of aptamer-based ALL cancer diagnostic probes and sensors have been proposed in published studies.

The Sgc8 aptamer is an active tumor-targeting ligand that binds to protein tyrosine kinase 7 (PTK7), a transmembrane receptor for cancer cells that is highly expressed in T cell ALL cell lines, such as CCRF-CEM cells and Molt-4 cells [67,68]. Subsequently, Sgc8 (88-mer) was further truncated and optimized to a 41-oligonucleotide form called sgc8c. It was shown that both sgc8 and sgc8c can bind with high affinity (Kd = 0.8 nM) and be specifically internalized into ALL-T cells [26,69,70].

Wu et al. [71]. developed a label-free fluorescent aptamer sensor for leukemia detection based on terbium(iii)-aptamer (Tb^3+^-apt). The Sgc8 aptamer makes the fluorescence of Tb^3+^ sensitive and forms a strongly fluorescent Tb^3+^-apt probe. The sensor can be used for ultra-sensitive and highly specific detection of ALL cells with a minimum detection limit of 5 cells per ml in binding buffer. In addition, the specificity and positivity rates of 100 clinical samples tested using the assay were 94% and 90%, respectively.

Bahreyni et al. [72] devised a novel method for detecting ALL cells based on a dual aptamer (Sgc8c and ATP aptamer)-functionalized graphene oxide (DAFGO) complex. The method works by internalizing the DAFGO complex into Molt-4 cells through Sgc8c as a molecular recognition probe, and then the complex releases FAM-labeled ATP aptamers to react with ATP in lysosomes to produce intense fluorescence. Li et al. [73] first used Sgc8-FITC to bind to silver decahedral nanoparticles (Ag10NPs) to form Ag10-Sgc8-FITC, and then used quencher-carrying strands to hybridize with Sgc8-FITC to form an Ag10NPs-based FRET (fluorescence resonance energy transfer) sensor. The sensor interacts with CCRF-CEM cells to achieve fluorescence imaging of the cells. Grechkin et al. [74] constructed aptamer-conjugated nanoparticles by binding Sgc8 to luminescent Tb-TCAS-doped silica nanoparticles and used fluorescence microscopy to demonstrate their ability to be used for ALL detection and imaging. In addition, they confirmed by cell viability assay that nanoparticles have no toxic side effects on cells, ensuring their potential application in ALL diagnostics.

Orit Jacobson et al. [75] used 2-step radiochemical synthesis with (18)F-labelled aptamer Sgc8 and validated it in vitro and in vivo in two cell lines, HCT116 and U87MG, which expressed high and low PTK7, respectively. the final in vivo and in vitro results were consistent with the high and low PTK7 expression in both cell lines, with U87MG xenograft tumors having tracer accumulation was much lower than that of HCT116 xenograft tumors. Quantification of PTK7 using (18)F-Tr-Sgc8 may be applicable to clinical translation and may assist in the selection and monitoring of appropriate therapies in the future.

Carmen Estévezgroup et al. [76] has developed biotinylated Sgc8 aptamers to be immobilized on the surface of neutrophil-encapsulated silica nanoparticles to identify cancer cells. This method can increase the detection sensitivity in flow cytometry analysis by a factor of 10 to 100 compared to standard methods. Ye et al. [77] designed an iodide-responsive copper-gold nanoparticle (Cu-Au) based nanoparticle that sensitively identifies target leukocytes. The aptamer-Cu-Au nanoparticles are capable of selective and ultrasensitive detection of cancer cells and can reach a detection limit of 5 cells in 100 μL of binding buffer.

Khoshfetrat et al. [78] immobilized thiolated Sgc8c aptamers in gold nanoparticle-coated magnetic Fe_3_O_4_ nanoparticles (Apt-GMNPs) and then ethidium bromide (EB) was embedded in the stem of the aptamer hairpin to provide a signal for the quantification of leukemia cancer cells. In the case scenario, the sensor showed a linear response over a wide dynamic range of 10 to 1 × 10^6^ cells/mL of leukemic cancer cells, exhibiting excellent detection limits.

Minimal residual disease (MRD) refers to a small number of leukemia cells that are left in the patient’s body during treatment or after remission, providing a high degree of independence as a prognostic factor for patients with leukemia. A biomimetic Multivalent Aptamer Nanoclimber functionalized microfluidic chip has been reported to be developed for the assessment and monitoring of ALL relapses by performing clinically applicable and highly sensitive MRD detection in T-cell ALL patients [79]. Li et al. [80] utilized a magnetic aptamer sgc8 probe and a rolling cycle amplification probe (RCA-sgc8 probe) for MRD detection in T-ALL patients during the peri-chemotherapy period. This strategy works by capturing CEM cells and then initiating RCA to generate DNA products that hybridize with molecular beacons to amplify the fluorescent signal on T-ALL cells.

Fluorescent organic dots (O dots) are a new contrast agent that is widely used for two-photon fluorescence imaging. Nevertheless, many of the currently developed two-photon absorption (TPA) O dots do not have a tumor-targeting component, making them unavailable for targeted tumor imaging applications. Yan et al. [81] developed a nanoprobe, including Sgc8c aptamer-mediated TPA O sites, that can be highly specific and rapidly internalized into ALL cells under the guidance of Sgc8c aptamers for effective targeted imaging in live cells or deep tissues.

A system of sgc8c aptamer conjugated magnetic beads and magnetic quartz crystal microbalance (QCM) was developed for the quantitative detection of ALL cells with a detection limit of 8 × 10^3^ cells/mL [82]. Shan et al. [83] also developed a QCM biosensor based on sgc8c, which efficiently captured CCRF-CEM cells by sgc8c and then silver-enhanced gold nanoparticle (AuNP) labeling to amplify the QCM signal for sensitive detection of ALL cells with a detection limit of 1160 cells/mL.

Hu et al. [84] prepared a photoelectrochemical (PEC) photosensitizer called PPIX-[BMIm] with aggregation-enhancing properties. Then, a label-free PEC cell sensor was established by electrodepositing Au NPs on the PPIX-[BMIm] aggregates and sequential assembly of sgc8 to achieve the detection of molt-4 cells with a lower limit of detection of 63 cells/mL. Muhammad’s team has developed a superior cancer screening package for early detection and diagnosis of ALL by detecting BCR-ABL1 mutated genes and CEA cancer biomarkers [85]. The results showed that the peak current of the catechol solution used as an electroactive probe on the biosensor correlated logarithmically and linearly with the target DNA and CEA antigen concentrations, with satisfactory detection limits of 1.5 pM and 0.26 pg mL^−1^, respectively.

#### 2.2.2. The Role of Aptamers in ALL Treatment

Moreover, sgc8c is gaining momentum in the treatment of ALL by combining with chemotherapeutic agents or nanoparticles.

Huang YF et al. [70] covalently linked Dox to Sgc8c and found that this Sgc8c-Dox coupling specifically killed CCRF-CEM cells. Mengting et al. [86] constructed Sgc8c-mediated DNA tetrahedral nanostructures (s-TDN) and found that s-TDN: Dox enhanced the toxicity to CCRF-CEM cells, but was less toxic to negative control cells. Zhang et al. [87] developed sgc8-based DNA dendritic nanostructures and delivered Dox through these aptamer-based DNA dendritic polymers with enhanced toxicity to ALL cells. Luo et al. [88] devised a drug carrier for targeting ALL by assembling Sgc8c on the surface of Au NP and loading Dox using a repeat sequence within hp DNA on the Au NP surface. They demonstrated that aptamer-functionalized nanoparticles containing drugs can kill CCRF-CEM cells more effectively compared to non-targeted cells. Zong et al. [89] proposed a complex nano-pharmaceuticals particle (BP NS@PEG@Sgc8@Dox) based on Sgc8 and black phosphorus nanosheets (BP NS). Due to the mediation of Sgc8 and the good optical properties of BP NS, targeted and synergistic chemophoto-therapeutic treatment of ALL was achieved, which provides a promising new strategy for the development of promising novel anticancer drugs.

Although Daunorubicin (Dau) is a commonly used clinical agent for the treatment of ALL, however, the toxic effects of Dau on the heart remain a major challenge for clinical application. Taghdisi et al. [90] introduced the sgc8-Dau complex as an effective system for targeted delivery of Dau to ALL T cells, which can effectively reduce drug side effects. Furthermore, a PA(polyvalent aptamers)-Dau-AuNPs complex was devised for specific delivery and internalization of Dau into Molt-4 cells. The PA-Dau-AuNPs showed the highest toxicity to Molt-4 cells compared to the control. The designed drug delivery system achieved efficient drug delivery, tumor targeting, pH-dependent drug release, and controlled delivery of Dau to tumor cells [91]. In another study, sgc8c-Dau-AuNPs complexes were designed and evaluated, which were capable of the pH-dependent release of Dau. The results showed that sgc8c-Dau-AuNPs were more cytotoxic to Molt-4 cells compared to Dau and sgc8c-Dau [92]. Taghdisi et al. [93] introduced sgc8c into the complex between Dau and Single-walled carbon nanotubes (SWNTs), and Dau achieved reversible and pH-dependent release from Dau-sgc8c-SWNTs, as well as the ability of Dau to be specifically delivered and internalized into Molt-4 cells to reduce side effects.

Cytarabine (Ara-C) is widely used to treat ALL, however, Dau’s clinical use is limited by its significant side effects. Fang et al. prepared Sgc8-targeted and glutathione (GSH)-responsive polymeric micelles (PCL-ss-Ara@Sgc8-BSA) that exhibited significant targeting as well as excellent GSH-responsive drug release behavior and antitumor effects on CCRF-CEM cells [94]. PCL-ss-Ara@Sgc8-BSA significantly enhanced the inhibition of tumor growth in xenograft-bearing mice with lower side effects and a higher survival rate compared to the control group. Similar to Ara-C, the therapeutic potential of vincristine (VCR) in ALL is limited by neurotoxicity. Duan et al. [95] developed a targeted liposome delivery complex (sgc8/VCR-Lipo) that significantly inhibited the proliferation of CCRF-CEM cells in vivo and reduced drug side effects. In conclusion, these studies provide good options for the development of novel aptamer-based targeted drug delivery systems.

Zhang et al. combined sgc8c with a high molecular weight synthetic semi-flexible polymer based on oligo (ethylene glycol)-modified poly-isocyano-peptide [96]. The material can effectively target ALL cells and inhibit their proliferation by inducing G0/G1 phase arrest. Therefore, this material is expected to be used for targeted ALL therapy.

Zhiyong Huang et al. [97] combined the aptamer Sgc8c and Combretastatin A4 (CA4) to develop three nucleic acid aptamer couples with different linkers: a phosphodiester-containing nucleic acid aptamer coupling, a disulfide-containing nucleic acid aptamer coupling and a carbamate-containing nucleic acid aptamer coupling to investigate the anti-cancer effects and drug release mechanisms of the different linkers, and ultimately found that the phosphodiester-containing linkers were more cytotoxic to cancer cells.

Intriguingly, only one aptamer, sgc-8, has been used for the diagnosis and treatment of all and has shown very good performance. This means another research could find more targeted aptamers for ALL. For the diagnosis of all, many researchers have combined sgc-8 with various nanomaterials, such as TB3+. DAFGO, AuNP, etc. By combining these assays with aptamers, the detection limit can be very low, which is of great practical significance for the early diagnosis of ALL patients. While in treatment of ALL by aptamers, researchers choose to couple aptamers with traditional chemotherapeutic drugs, enhancing the toxicity to target cells and weakening the damage to normal cells. In addition, nanoparticles such as black phosphorus are combined with photothermal reactions to treat ALL. It could be said that the research of sgc-8 has been very well developed in the diagnosis and treatment of ALL, and it is now the focus of researchers to advance these research results to the clinical level.

### 2.3. Aptamers in CML

CML is a malignancy formed by the clonal proliferation of bone marrow hematopoietic stem cells [98]. Most patients with CML have the Philadelphia (Ph) chromosome, which is the product of a t (9; 22) translocation between chromosomes 9 and 22. During this translocation, the ABL fragment of the proto-oncogene on chromosome 9 translocate to chromosome 22 and fuse with the BCR gene, forming the chimeric fusion gene BCR-ABL [99]. Oncogene-specific gene therapy is a highly promising strategy for the treatment of CML.

Ping et al. developed an aptamer that specifically targets the BCR-ABL gene and found that the aptamer-siRNA complex significantly induces apoptosis in the myeloid leukemia cell line K562 cells, providing a reference for gene therapy in CML patients [100].

The use of tyrosine kinase inhibitors (TKI) can significantly improve CML outcomes and prolong survival, but resistance to TKI has become a serious challenge in the treatment of myeloid leukemia. NOX-A12 is an RNA oligonucleotide in L-configuration (Spiegelmer) that binds and neutralizes the chemokine CXCL12 with high affinity and specificity, The results of Weisberg et al. showed that the combination of NOX-A12 and TKIs was able to block CXCL12-induced migration of BCR-ABL-positive leukemia (e.g., CML) cells more effectively in vitro to promote the treatment of cancer, and the same results were obtained in vivo experiments in CML model mice [101].

There are still relatively few studies on aptamers in CML, mainly on the use of aptamers as a targeting tool for CML cells using siRNA to induce apoptosis to achieve therapeutic effects, or aptamer-specific binding to target cells can effectively inhibit chronic myeloid leukemia cell migration. Currently, there is still a large gap in the diagnosis and treatment of CML with aptamers. Although the treatment of CML is well established, with the low price of aptamers, the diagnosis and treatment of aptamers in CML still have the potential for continued research.

### 2.4. Aptamers in CLL

CLL is the most widespread type of leukemia in Western adults (accounting for approximately 25% of adult leukemias), with a median age at diagnosis of 72 years and a higher incidence in males [102,103,104,105]. Common symptoms are peripheral blood lymphocytosis, lymph node enlargement, hepatosplenomegaly, bone marrow failure, autoimmune hemolytic anemia, and thrombocytopenia [106,107]. CLL is a heterogeneous disease that can be divided into indolent and aggressive forms based on its clinical course and response to treatment. Some patients survive longer without symptoms for decades after diagnosis or without the disease progressing to severe disease, while others progress rapidly to a high-risk state requiring immediate treatment after diagnosis and may eventually die due to complications from treatment or disease progression. However, the clinical status of most CLL patients falls somewhere in between these two conditions requiring early diagnosis or treatment [105,108,109]. Therefore, the aptamer can be very helpful as a potential clinical diagnostic or therapeutic tool for patients with CLL.

The amount of eEF1A1/eEF1A2 was quantified in the lymphocytes of 46 CLL patients versus normal controls. Dapas et al. [110] found elevated eEF1A1/eEF1A2 in CLL lymphocytes. In addition, eEF1A1 levels were higher in patients who died compared to survivors. Therefore, eEF1A1 may serve as a prognostic marker and a therapeutic target for CLL. GT75 aptamer, which contains the GT (guanosine-thymidine) repeat T(GTTT)18GT (75 nucleotides total length). This aptamer can bind eEF1A1 in a variety of human cells [111,112,113]. The Dapas team used GT75 to cause reduced viability/autophagy stimulation of MEC-1 (CLL cells) and downregulation of tumor growth in vivo.

CXCL12 is a chemokine that supports the survival of CLL cells in the bone marrow and lymph node microenvironment by interacting with CXCR4 to promote the homing and retention of hematopoietic and immune cells and their trafficking [114,115]. In a phase IIa study, treatment with NOX-A12 was found to improve the pharmacodynamic effect of CLL cells effectively [116]. Hollenrigel et al. showed that by inhibiting CXCL12 with NOX-A12, they could modulate the tumor microenvironment, thereby mobilizing leukemic cells to the peripheral blood and making CLL cells more susceptible to cytotoxic drugs [117]. The research on aptamer therapy for CLL is still in its initial stage, and researchers have found higher levels of EEF1A1 in lymphocytes of CLL patients, so they have screened it as a therapeutic target to obtain aptamers for CLL. The aptamer in CLL is a Protein-SELEX progress, and perhaps using Cell-SELEX to screen for cell lines commonly used in CLL may lead to new targets for CLL therapy, which may be of great help in the treatment of CLL.

In leukemia diagnosis and treatment, aptamers have been studied in a considerable number (Table 2), in which they contribute to the early diagnosis of leukemia and also serve as drug delivery tools for targeted therapy, enhancing the toxicity of various chemotherapeutic drugs to target cells and reducing the damage to normal tissues of the patient’s body, indicating that there is great potential for the application of aptamers in the clinical diagnosis or treatment of leukemia. However, there are still many problems in the research of aptamers in leukemia, firstly, there are abundant studies in AML and ALL, but there is still a lack of sufficient studies in CML and CLL, leaving a big gap that needs to be further promoted. Secondly, among these aptamers, only AS1411 and NOXA-12 have been promoted in clinical trials, while other aptamers still need to be tested in clinical trials. Meanwhile, the development of aptamers as early diagnostic reagents for leukemia can be considered. Aptamers are low-cost and very sensitive for leukemia diagnosis in combination with various biomaterials, and it is valuable to develop low-invasive diagnostic reagents for leukemia.

## 3. Aptamers in Lymphoma

Lymphomas are hematologic malignancies originating from different types of lymphocytes, mainly located in lymph nodes or other lymphatic structures, and are considered solid tumors of the immune system [120,121]. Malignant lymphoma is now the most prevalent hematological neoplasm and its pathological types can be divided into non-Hodgkin’s lymphoma (NHL) and Hodgkin’s lymphoma (HL). The latter can be further divided into B cell origin and T/NK cell origin according to its source [122,123,124]. Currently, aptamer-mediated targeted therapy and early diagnosis offer a promising new approach to lymphoma research (Table 3).

Studies show that CD30 protein is overexpressed in anaplastic large cell lymphoma (ALCL) and classical Hodgkin’s lymphoma(cHL) [125,126]. ALCL is a separate type of NHL. Parekh et al. generated ssDNA aptamers (C2) that bind CD30 with high affinity and specificity by a hybrid SELEX approach, and further optimized them to produce shorter truncated variants (C2NP,31nt) with 50-fold higher affinity [127]. They found that C2NP polymers could induce apoptosis in ALCL cells by CD30 receptor oligomerization followed by activation of downstream signals. Xiao Luo et al. developed PEG-PLGA NPs encapsulated with Dox and modified with C2NP to prepare targeted formulations [128]. The release time of the drug was effectively increased in the targeted formulation and accumulated more in ALCL cells than in non-targeted cells. This demonstrates the great potential of using C2NP-functionalized nanoparticles for the treatment of ALCL. Therefore, Chen Run et al. [129]. modified straight and twisted DNA nanotubes with C2NP and loaded Dox, in which the interaction of C2NP with its receptor CD30 upregulated p53 expression and led to apoptosis of K299 cells (ALCL cell line). Zhao et al. exploited the aberrant expression of CD30 and anaplastic lymphoma kinase (ALK) genes in lymphoma cells by combining ALK siRNA and RNA-based CD30 aptamer probes into nano-sized polyethyleneimine-citrate carriers to construct nanocomplexes that specifically silenced ALK gene expression in lymphoma cells, causing growth arrest and apoptosis [130]. In addition, a multifunctional aptamer-nanomedicine (Apt-NMed) containing not only CD30-specific aptamer and ALK-specific siRNA but also loaded with Dox was developed to enable targeted chemotherapy and synergistic gene therapy for ALCL. The Apt-NMed-mediated treatment not only significantly improved the survival of the mice compared to the control group but also reduced the side effects [131]. Zeng reported a CD30 aptamer-modified protamine nanomedicine containing a dsDNA/drug that effectively killed lymphoma cells as well as an ALK-specific siRNA, and importantly, the complex was virtually nontoxic to off-target cells [132]. CD30 aptamer has excellent performance in the treatment of ALCL, which can effectively increase the retention time of the drug in the target cells and prolong the release time of the drug, as well as specifically target siRNA to ALCL cells to inhibit ALK gene expression, leading to cell growth arrest and death, and has almost no toxic effects on non-targeted cells, these results suggest that CD30 may be a potential tool for targeted therapy against ALCL.

Based on the property that CD19 is overexpressed in the majority of human B-cell tumors, Yan Hu et al. identified the CD19 aptamer (LC1), a 59-nucleotide ss DNA that binds recombinant CD19 protein with high affinity (Kd = 85.4 nM) [133]. LC1 was shown to specifically bind CD19-positive lymphoma cells (Ramos and Raji) and selective delivery of Dox was achieved in vitro by constructing LC1-Dox.

Mallikaratchy et al. used Ramos cells to identify TD05 aptamers that selectively bind membrane-bound immunoglobulin mu heavy chains, a target that is a major component of the B-cell receptor expressed in Burkitt’s lymphoma and could serve as a biomarker for Burkitt’s lymphoma [134,135,136]. A few years later, Mallikaratchy’s group prepared TD05 aptamer-engineered polymorphs that reacted with the B-cell receptor and demonstrated B-cell specificity in lymphoma cell lines and fresh clinical leukemia samples [137].

The B-cell activating factor (BAFF) receptor (BAFF-R) is overexpressed in B-cell malignancies such as NHL and may serve as a potential biomarker for tumors. Zhou et al. used in vitro SELEX to select 2’-F-modified RNA aptamers with a high affinity for BAFF-R that were able to block BAFF-mediated B cell proliferation and internalize it into B cells by binding effectively to BAFF-R [138]. Subsequently, Zhou et al. described a BAFF-R aptamer-siRNA delivery system that specifically delivered STAT3 siRNA to various B-cell lines, resulting in gene silencing of mesangial STAT3 mRNA [139].

Opazo et al. reported a DNA aptamer (C10.36) with an unconventional G-quadruplex structure that binds with high affinity and specifically enters Burkitt lymphoma cells and can be used as an efficient tool for the specific delivery of different cargoes to tumor cells [140]. Through further studies, Tonapi et al. found that some lymphoma cells display a spliceosome complex (SSC) on their surface, consisting of at least 13 core components, and are binding targets for C10.36. Furthermore, the binding of C10.36 to lymphoma cells triggers SSC internalization, leading to global changes in variable splicing patterns and ultimately cell death [141].

CD20 is a target of B cells, and Haghighi et al. used Cell-SELEX on recombinant HEK293T-CD20 cells to identify an aptamer named AP-1 that binds CD20 with high affinity (96.91 ± 4.5 nM), promising for diagnostic and therapeutic applications in lymphoma [142]. A novel anti-lymphoma nano-drug CD20 aptamer-RBCm@Ag-MOFs/PFK15 (A-RAMP) was designed and prepared [143]. The results showed that A-RAMP could effectively target B-cell lymphoma and exert synergistic antitumor effects with Ag^+^ and FPK15 by reprogramming aerobic glycolysis.

Zümrüt et al. recently introduced a SELEX variant called “Ligand-Guided-Selection” (LIGS) that recognizes and selects for specific aptamers of known cell surface proteins [144]. The aptamer identified by LIGS has a higher specificity for the target ligand than the typical Cell-SELEX. Using LIGS, they identified an aptamer that binds IgM on target cells, named R1. Subsequently, they designed a truncated variant of R1 with higher affinity (R1.2) that specifically binds IgM-positive human B lymphoma BJAB cells at physiological temperature. Despite the high specificity of the LIGS-identified aptamers, their optimized and still unsatisfactory affinity prevented the use of the aptamers for clinical translational applications. Batool et al. formed high-affinity variants by dimerizing R1.2, and importantly, binding experiments using CRISPR/Cas9 on IgM knockout Burkitt’s lymphoma cells verified that dimeric R1.2 did not affect the original specificity [145]. Thus, LIGS-identified aptamers can be re-optimized into dimeric aptamers with high affinity and specificity, which provides a new strategy for developing clinical diagnostic and therapeutic aptamers. LIGS provides a new screening method with higher efficiency and specificity than the previous hybrid screening of Protein-SELEX and Cell-SELEX, but the main problem is the low affinity, which may need to be optimized by introducing some external modifications.

Although natural killer (NK) cells are an integral part of the immune system, they lack cellular targeting vectors for targeted cancer immunotherapy under normal circumstances. Yang et al. anchored CD30-specific aptamers to the cell surface to generate aptamer-engineered NK cells (ApEn-NK), and targeted NK cells guided by aptamers triggered higher apoptosis/mortality in lymphoma cells, thus providing a promising new approach for NK cell-targeted immunotherapy [146].

Parekh et al. identified an ssDNA aptamer (PS1NP) with high affinity (5 ± 0.5 nM) and specific binding to HL based on Cell-SELEX, which selectively detects HL cells in cell mixtures and even in complex biological media, demonstrating the potential of this aptamer to be used as a vector for targeted drug delivery and the potential of the aptamer to detect HL cells [147].

In addition, many nanomaterial-aptamer complexes have also been designed for tumor diagnosis in lymphoma. Slyusarenko et al. created an AuNP aptamer sensor analysis system to quantify plasma CD30 small cell extracellular vesicles [148]. The developed method can differentiate between healthy donors and cHL patients, providing a promising new method for monitoring cHL patients.

An ultrasensitive nanopore sensor based on aptamer recognition and signal amplification for label-free detection of aerosolized cancer cells was developed for the first time by Xi et al. By introducing robust, one-step enzymatic cycling amplification in the aerosol nanopore system, Ramos cells down to 5 cells in number can be identified in this assay [149]. Fazlali et al. introduced new label-free electrogenerated chemiluminescence aptasensors for the detection of lymphoma cells by exploiting the binding of aptamers to CD20 on the surface of B-cell membranes and the accumulation of the positive charges of a nanocomposite on the negatively negative-charge charged of the aptamer phosphate backbone [150]. The complex achieved enhanced sensitivity detection of B-lymphoma cells with a detection limit of 31 cells/mL. In addition, a novel aptamer biosensor for cancer cell determination based on ultrasensitive electrochemical detection has been reported [151]. The cancer cells are first recognized and bound by the aptamer, and then the cell-aptamer mediates an alkaline phosphatase-catalyzed silver deposition reaction that can be detected by electrochemical detection. This method exhibits excellent selectivity in Ramos cell identification with detection limits as low as 10 cells. In conclusion, the above strategies demonstrate the promising potential application of aptamers in the early diagnosis of lymphoma.

As a hematologic tumor with various subtypes, lymphoma has the highest incidence among hematologic tumors, and therefore many aptamers have been created for targeting and diagnosing various subtypes of lymphoma, and these aptamers have contributed to the future treatment of various lymphomas. For example, aptamers targeting CD30 have been richly studied in the diagnosis and treatment of ALCL and cHL, and researchers have loaded these aptamers with chemotherapeutic drugs and siRNAs to achieve targeted therapy, resulting in many potential targeted therapeutic regimens. Many studies have combined CD20 aptamers with various materials to construct diagnostic tools for lymphoma with high sensitivity and selectivity. In conclusion for this part, aptamers have shown sufficient potential for application in the treatment of lymphoma, and further optimization is needed for further development.

**Table 3:** A summary of aptamers that have been identified to lymphoma. Kd = dissociation constant.

Name	DNA/RNA	Type of SELEX	Target Cell	Target Molecule	Tested	Kd	Conjugated Molecule	Application	Reference
C2NP	DNA (31nt)	Hybrid SELEX	K299	CD30	In vitro			Growth inhibition, apoptosis induction	[127]
			K299		In vitro; In vivo		ALK siRNA, Dox	Combined cell-selective chemotherapy and oncogene-specific gene therapy. Apoptosis induction and growth inhibition in vitro and prolongation of survival in mice in vivo.	[131]
			K299, L428		In vitro		PEG-PLGA NPs, Dox	Apoptosis induction and growth inhibition	[128]
			K299		In vitro		S-DNT, T-DNT, Dox	Apoptosis induction and growth inhibition	[129]
			K299		In vitro		NK cells	Growth inhibition, apoptosis induction. Immunotherapy	[146]
	RNA		K299		In vitro		ALK siRNA	Growth arrest and apoptosis	[130]
	RNA		K299		In vitro		dsDNA/Dox ALK siRNA	Combined cell-selective chemotherapy and oncogene-specific gene therapy. Growth inhibition	[132]
PS1NP	DNA	Cell-SELEX	HDLM2, K299		In vitro	5 ± 0.5 nM		A carrier for in vivo targeted drug delivery	[147]
LC1	DNA (59-mer)	Protein-SELEX	Ramos, Raji	CD19	In vitro	85.4 nM	Dox	Growth inhibition	[133]
TD05	DNA	Cell-SELEX	Ramos	IGHM, mIgM	In vitro			Identification of potential markers	[134,137]
R1.2	DNA	LIGS	BJAB	mIgM	In vitro	35.5 ± 8.94 nM (4 °C), 65.6 ± 5.88 nM(37 °C)		Identification of potential markers	[144,145]
R-1	RNA	in vitro SELEX	Jeko-1	BAFF-R	In vitro	47 nM	STAT3 siRNA	Growth inhibition	[139]
C10.36	DNA (36-mer)		Ramos	SSC	In vitro			Growth inhibition	[142]
AP-1	DNA	Cell-SELEX	HEK293T -CD20	CD20	In vitro	96.91 ± 4.5 nM			[142]
			Raji		In vitro; In vivo		Ag-MOFs, PFK15, RBCm	Apoptosis induction and growth inhibition in vitro and prolongation of survival in mice in vivo.	[143]

## 4. Aptamers in Multiple Myeloma

A malignant disease of plasma cell (PC) origin, MM causes approximately 1% of all cancer-related deaths and 13% of hematological malignancies each year, mainly affecting older adults with a median age of 69 years [152]. MM presents with abnormal proliferation and accumulation of bone marrow PC and excessive production of monoclonal immunoglobulins, often accompanied by multiple osteolytic lesions, hypercalcemia, anemia, and renal injury. Currently, the development of chemotherapy, autologous stem cell transplantation, proteasome inhibitors, immunomodulators, nucleic acid analogs [153,154,155], and their combination therapies have effectively prolonged the survival time of new cases of MM, but for most patients, MM remains an incurable disease [156,157]. As biomedicine continues to advance, aptamers show promising clinical prospects in the treatment of MM (Table 4).

B-cell maturation antigen (BCMA) is present on the cell surface of late-stage normal B lymphocytes and is highly expressed in malignant plasma cells of MM patients, where it plays a role in cell survival [158,159]. apt69.T is the first RNA aptamer targeting BCMA that inhibits the APRIL-dependent BCMA downstream NF-kB pathway and rapidly internalizes it in MM cells [160]. In addition, the aptamer binds to microRNA-137 (miR-137) and anti-miR-222, showing a high potential to inhibit tumor cells.

Annexin A2 (ANXA2) is a member of a protein superfamily that is closely associated with malignant tumorigenesis, proliferation, invasion, and metastasis [161,162,163] and is highly expressed in MM patients [164,165]. Zhou’s group used the Protein-SELEX method to identify the ss DNA aptamer (wh6) that binds ANXA2 protein with high affinity and specifically binds MM cells [166]. wh6 inhibits ANXA2-induced MM cell proliferation by disrupting the adhesion of MM cells to ANXA2.

Activation of the hepatocyte growth factor (HGF)/c-met pathway is closely related to the pathogenesis of MM, and therefore blocking this pathway is a new strategy for the treatment of MM. The DNA aptamer SL1 binds c-met with high specificity and affinity and inhibits HGF/c-met signaling in SNU-5 cells [167]. Through the study of SL1 in MM, Yibin Zhang et al. found that SL1 could target c-met in MM and inhibit MM cell growth, migration, and adhesion in vitro [168]. Furthermore, SL1 synergistically inhibited the proliferation of CD138^+^ primary MM cells with bortezomib.

The transmembrane glycoprotein CD38, which is widely used to detect PC and diagnose MM, is a promising biomarker for targeting MM therapy. Wen et al. identified a CD38-specific ssDNA aptamer (#1S) that can target MM cells with high affinity [169]. The #1S aptamer is loaded with Dox by non-covalent embedding, and the aptamer-drug complex is specifically delivered to MM cells, and Dox is efficiently released intracellularly under a unique pH control mechanism to achieve selective inhibition of MM tumors [170].

Dai et al. used Cell-SELEX to identify ss DNA aptamer TY04, which binds specifically to MM cells and induces cell cycle arrest by upregulating CDK1 and cyclin B1 expression and downregulating γ-microtubulin expression, leading to cell growth inhibition [171].

Cell adhesion-mediated drug resistance (CAM-DR) in bone marrow is the basis for MM cell dissemination and survival. In phase I/II clinical trials, data suggest that CXCL12 is a potent target for antagonizing CAM-DR in MM. The combination of NOX-A12 with bortezomib-dexamethasone resulted in the re-sensitization of MM cells to anti-myeloma drugs, suggesting that NOX-A12 could serve as an effective add-on agent to antagonize myeloma CAM-DR [172].

In conclusion, there is an abundance of aptamers for multiple myeloma, some of which bind to surface proteins and block certain pathways to organize the proliferation, invasion, and migration of MM cells, while others work in combination with chemotherapeutic agents such as DOX. Although there are many aptamers related to MM, these studies did not dig deeper to promote the application of these aptamers in the diagnosis and treatment of MM. Perhaps researchers can consider combining these aptamers with various nanomaterials to build MM diagnostic reagents to facilitate early screening of MM patients to improve survival rates.

**Table 4:** A summary of aptamers that have been identified to MM. Kd = dissociation constant.

Name	DNA/RNA	Type of SELEX	Target Cell	Target Molecule	Tested	Kd	Conjugated Molecule	Application	Reference
apt69.T	RNA (50-mer)	Cell-SELEX, Cell-internalizing SELEX	U266, H929	BCMA	In vitro	79.4 nM	miR-137, anti-miR-222	Growth inhibition	[160]
wh6	DNA (80 nt)	Protein-SELEX	MM.1R, MM.1S, ARP-1, ANBL-6 and RMPI-8226 cell	ANXA2	In vitro; In vivo	8.75 ± 1.26 nM		Adhesion and proliferation inhibition of MM cell lines in vitro and tumor targeting in vivo.	[166]
TY04	DNA	Cell-SELEX	MM.1S, NCI-H929, KM3, OPM2		In vitro			inhibits the growth of multiple myeloma cells via cell cycle arrest	[166]
SL1	DNA (50-mer)	Cell-SELEX	CD138^+^ cells (MM.1S, ARP-1)	c-met	In vitro; In vivo. Ex vivo	135.6 nM (MM.1S), 237.1 nM (ARP-1)		Inhibition of MM cell growth, migration and adhesion in vitro and tumor targeting in vivo	[168]
#1S	DNA	Hybrid SELEX (cell-based SELEX and protein-based SELEX)	CD38^+^cells (MM.1S, RPMI8226, MM1R, NCI-H929, Dox40)	CD38			Dox	Growth inhibition and apoptosis induction in vitro and prolongation of survival in mice in vivo.	[169,170]

## 5. Conclusions and Discussion

In this review, we summarize the nucleic acid aptamers currently associated with hematologic malignancies, including their research progress, and their pros and cons of existing aptamers in various types of hematological tumors. In this way, we provide researchers with assistance in the subsequent further study of these hematological malignancy aptamers.

Hematological malignancies have many subtypes and high heterogeneity, and currently, usually rely on chemotherapy and radiotherapy to control tumor progression. However, chemotherapy and radiotherapy lead to unavoidable side effects, including toxicity to normal tissues and high recurrence rates. Therefore, targeted therapy for hematological malignancies is an extremely promising area of research. Recently, emerging affinity molecular aptamers have emerged not only as therapeutic agents but also as effective vehicles for the targeted delivery of anticancer drugs or RNAs into cancer cells to exert their anticancer activity while minimizing side effects on healthy tissues or cells.

**Figure 2:**
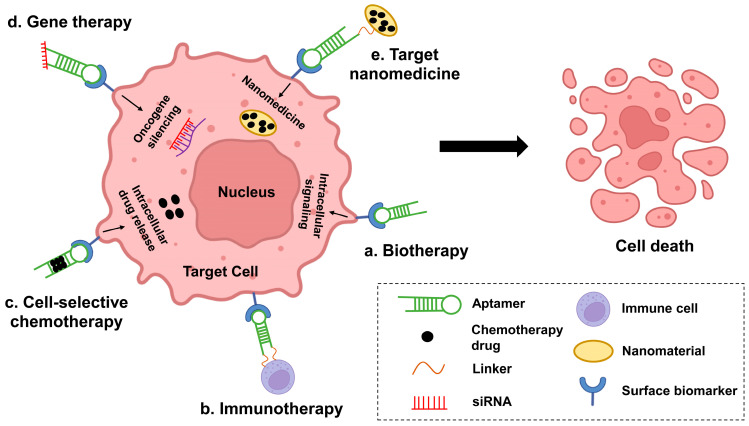
Aptamer-mediated five major tumor treatment options. (**a**). Biotherapy: Aptamers recognize and bind to cell membrane surface targets, internalize into cells, and exert anticancer activity by activating or inhibiting relevant intracellular signaling pathways. (**b**). Immunotherapy: By coupling the aptamer to immune cells, immune cells target recognition and bind to target cells for immune activity. (**c**). Cell-selective chemotherapy: Aptamer-drug couples recognize and bind to target membrane proteins of cancer cells, and after internalization, chemotherapeutic drugs are released intracellularly for efficient activity. (**d**). Gene therapy: Aptamer-siRNA couples recognize and bind to the target membrane proteins of cancer cells, and after entering the cells siRNA exerts anti-cancer activity by silencing the expression of oncogenes within the cells. (**e**). Target nanomedicine: The aptamer-nanomedicine recognizes and binds to the cellular target, which is then internalized by target-mediated endocytosis, and after internalization, the nanomedicine is transported to the lysosome, and finally the drug is out and exerts its drug activity.

In this case (Figure 2), aptamers have become a useful tool for the development of novel therapies (targeted therapies). Compared to conventional targeted drug antibodies, aptamers have little immune response, can be selected for a wider range of targets, and even have specific targeting ligands with similar or greater binding affinity, thus providing more options for the diagnosis and treatment of cancer. They can distinguish cancer cells from healthy cells with high sensitivity and specificity. Given the complex pathogenesis of hematologic malignancies, the development of broadly targeted and highly specific aptamer-based therapeutic approaches is valuable. In addition, aptamers are relatively easy to penetrate solid tumor tissue because of their small molecular weight. They are produced in vitro and relatively lower cost than monoclonal antibodies. Recent years, nanotechnology has been introduced to improve the utility of aptamers. Aptamers confer targeting properties to nanoparticles as well as reduce their immunogenicity, while nanoparticles prolong the half-life of aptamers in vivo and reduce their enzyme digestion. In addition, aptamer-nanoparticles can achieve more sustained and specific release of anticancer drugs, thereby reducing the drug dose usage. Thus, aptamer-nanoparticle-drug combinations can provide promising therapeutic options for a variety of diseases. In the last decades, applications of aptamers are growing quickly in both the treatment and diagnosis of hematologic malignancies. As shown in Figure 2, current aptamer-mediated relevant hematologic oncology treatment options fall into five major categories: biotherapy, cell-selective chemotherapy, oncogene-specific gene therapy, target nanomedicine, and immunotherapy. As aptamer therapy protocols mature, they may be of great help in the treatment of hematologic tumors.

Nevertheless, there are still some hurdles to overcome before aptamer technology can be more widely accepted as a cancer treatment such as antibodies. Firstly, a major hurdle in aptamer development is clinical translational application. Few aptamers have entered late-stage clinical trials due to difficulties in targeting specificity in vivo due to their oligonucleotide biology, susceptibility to rapid enzyme digestion in the bloodstream in vivo, and difficulty in identifying affected intracellular pathways after internalization. Compared to antibodies, aptamers still suffer from lower affinity than antibodies because SELEX screening of aptamers is still mostly performed in an artificial environment as opposed to a physiological one. It should be noted, however, that effective solutions have been proposed to overcome some of these obstacles. Studies have shown that the use of chemically modified nuclease-resistant aptamers or coupling with nanomaterials is an effective way to prevent nuclease degradation of aptamers [173,174,175]. For example, the use of fluorine, amino, or methoxy functional groups to replace the 2′OH of the RNA sugar backbone or the use of locked nucleic acids improves the resistance of aptamer nucleases [176,177]. PEG-coupled aptamers or aptamers coupled to cholesterol molecules can also improve their half-life and biostability in vivo to some extent [178,179]. Moreover, the expensive and time-consuming SELEX process with a low success rate has hindered the clinical application of aptamers, so there is an urgent need for high-throughput selection methods to screen for aptamers with clinical potential. Secondly, while many of the studies cited in this review have identified specific targets for the discussed aptamers, the biggest challenge in generating aptamers via Cell-SELEX is target identification [180,181]. It is well known that cell membrane-associated proteins are difficult to isolate and that aptamer targets are not always the most abundant proteins in the sample, making downstream proteomic identification methods difficult. Although some progress has been made in the use of chromatographic methods [182] or detailed proteomic analysis [183] for aptamer target identification, it is still difficult to be widely used. Therefore, a more in-depth study of the structural basis of known aptamer-target binding complexes may help guide the future development of target identification as well as aptamer-based targeted therapies. Thirdly, another limitation of the applicability of aptamers is their inability to cross the plasma membrane by passive diffusion. However, several strategies have been applied to overcome this challenge, such as the use of nanoparticles [184], liposome transfection [185], or delivery via viruses or plasmids [186]. Finally, although great progress has been made in the research of aptamers for oncology applications, continuous improvement is still needed in terms of delivery rate, targeting efficiency, cycle time, and affinity. The combination of aptamer and drug as well as the modification of aptamer on nanocarriers still needs to be improved. In addition, further development of aptamer synthesis technologies is needed, such as the production of shorter sequences, lower cost, better tumor penetration, and more blood-stable aptamers to facilitate use in cancer diagnosis and treatment. At the same time, the lack of adequate production facilities and skilled personnel with relevant educational backgrounds is a hurdle to overcome compared to the antibodies of a mature commercial infrastructure.

All in all, aptamers have opened an attractive and promising field of targeted therapeutic medicine in cancer diagnostics and treatment. As SELEX technology and chemical modification methods continue to advance, more new aptamers with excellent biological functions will emerge, and we believe that aptamers will certainly play an increasingly important role in future oncology applications.

## Figures and Tables

**Table 1:** A comparison between aptamers and antibodies.

	Aptamers	Antibodies
Composition	DNA or RNA (A, G, T, U, C)	Protein (Amino acid)
Size	Small (1–2 nm; <30 kDa)	Large (~15 nm; ~150 kDa)
Immunogenicity	Low	High
Binding affinity	High (nanomolar to picomolar range)	High (nanomolar to picomolar range)
Targets	Widely, from ions and small molecules to whole cells and live animals	Proteins mainly
Target Size	≥600 Da	≥60 Da
Synthesis time	Short (2–8 weeks)	Long (more than 6 months)
Reproducibility	None or little variation between batches	Wide variation between batches
Manufacturing Process	In vivo	In vitro
In vivo half-life	Short	Long
Cost	Cheap	Expensive
Modification	Easy	Hard
Stability	High (stable at a wide range of temperatures; reversible denaturation)	Low (susceptible to high temperatures and pH changes. irreversible denaturation)
Tissue penetration/kidney filtration	Fast	Slow
Toxicity	Low	High

**Table 2:** Summary of aptamers that have been identified to leukemia. Kd = dissociation constant.

Disease	Name	DNA/ RNA	Type of SELEX	Target Cell	Target Molecule	Tested	Kd	Conjugated Molecule	Application/Mechanism	Reference
AML	ZW25; CY30	DNA (66-mer)	Protein-SELEX	CD123+ cells	CD123	In vitro; In vivo	29.41 nM; 15.38 nM	Dox (By ZW25 mediated)	Inhibition of AML cells growth in vitro and prolongation of survival in mice in vivo	[43]
	SS30 (thioaptamer from CY30)	DNA (66 bp)		CD123^+^ cells		In vitro; In vivo	39.1 nM for CD123 peptide and 287.6 nM for CD123 AML cells		Selective inhibition of proliferation of AML cells in vitro and prolongation of survival in mice in vivo via JAK2/STAT5 signaling pathway	[44]
								sgRNA-targeting sequence		[45]
	#1 (#1-F)	DNA (77-mer)	Hybrid-SELEX	HEL cells	CD117	In vitro	4.24 nM	MTX	specifically inhibits AML cell growth and induces cell cycle arrest in the G1 phase	[52]
	K19	DNA (50-mer)	Cell-SELEX	NB4	Siglec-5	In vitro	12.37 nM		Detection of low concentrations of AML cells	[118]
								two “lock” sequences and two G-rich sequences	Building a luminescence sensing platform to detect Siglec-5	[119]
	AB3	DNA (59nt)	Protein-SELEX	HL-60, Jurkat, Ramos	OFA/iLRP	In vitro	101 nM	Dox	Selective delivery of Dox to OFA/iLRP-positive AML cells	[54]
	S30-T1	DNA	Paired cell-based SELEX	CD33 transfected-HEK293T, HL-60	CD33	In vitro; In vivo	~43 nM	Dox	Highly identifiable AML cells in vitro and in vivo. Specifically recognize and inhibit HL-60 cell proliferation by arresting the cell cycle at the G2 phase in vitro.	[56]
	AS1411	DNA (26-mer)		NB4, Kas-1,HL60	Nucleolin	In vitro; In vivo		AuNP, anti-221	AS1411 and anti-221 synergistically suppress AML cell growth via targeting key molecules involved in NCL/miR-221/NFκB/DNMT1 pathway in vitro and in vivo	[61]
	KGE02	DNA (76-mer)	whole-cell SELEX	MLL-AF9 RAS (MA9Ras) AML cells	MLL-AF9	In vitro	37.5 ± 2.5 nM		A DNA aptamer specific to AML cells was developed and characterized for future drug-aptamer conjugates.	[58]
ALL	Sgc8 (Sgc8c)	DNA	Cell-SELEX	CCRF-CEM	PTK7	In vitro, Ex vivo	0.8 nM	Tb^3+^	Cellular detection and early diagnosis of ALL	[71]
						In vitro, Ex vivo		DEAS/PMMA-co-MAA	Precision targeting and imaging in ALL cells	[81]
						In vitro		Ag 10 NPs	Cellular detection and imaging	[73]
						In vitro, In vivo		18F	Cellular detection and imaging	[75]
						In vitro		silica	Cellular detection	[76]
						In vitro		Cu-Au NPs	Cellular detection	[77]
						In vitro		GMNPs	detection of leukemia cells	[78]
						In vitro		MBs	Selective collection and detection of ALL cells	[82]
						In vitro		APBA-AuNPs	Cellular detection	[83]
						In vitro		BP NS, Dox, PEG	Growth inhibition, targeted and synergetic chemophotothermal therapy of ALL	[89]
						In vitro		Dox	Growth inhibition of ALL cells	[70]
						In vitro		Tetrahedron DNA, Dox	More cytotoxic to ALL cells	[86]
						In vitro		Combretastatin A4	More cytotoxic to ALL cells	[75,97]
						In vitro; In vivo		PCL-ss-Ara, BSA	Inhibition of ALL cells growth in vitro and prolongation of survival in mice in vivo	[94]
						In vitro		Au NP, Dox	Growth inhibition of ALL cells	[88]
						In vitro		DNA dendrimer, Dox	Growth inhibition of ALL cells	[87]
						In vitro		PIC	Inhibition of AML cells proliferation by G0/G1 phase arrest	[96]
						In vitro		LIPO, VCR	More cytotoxic to ALL cells	[95]
				Molt-4		In vitro		Dau	Growth inhibition of ALL cells	[90]
						In vitro		AuNPs, Dau, AS1411	Growth inhibition of ALL cells	[91]
						In vitro		AuNPs, Dau,	More cytotoxic to ALL cells	[92]
						In vitro		SWNT, Dau	More cytotoxic to ALL cells	[93]
						In vitro		ATP aptamer, DAFGO	Detection and early diagnosis by complexes being efficiently internalized into ALL cells and inducing intense fluorescence emission	[72]
						In vitro		PPIX-[BMIm], Au NPs	Cellular detection	[84]
CML				K562		In vitro		siRNA	The aptamer-siRNA compound can significantly induce K562 cell apoptosis	[100]
	NOX-A12	RNA (45-mer)		BCR-ABL-positive leukemia cells	CXCL12 (SDF-1)	In vitro; In vivo			Combined use of targeted kinase inhibition and NOX-A12 for treatment. Cell migration	[101]
CLL				CLL cells from peripheral blood samples		In vitro			NOX-A12 enhances the cytotoxicity of anticancer drugs by mobilizing leukemia cells to peripheral blood through inhibition of SDF-1. CLL migration and drug resistance	[117]
	GT75			MEC-1	eEF1A1	In vitro; In vivo		siRNA (siA1)	MEC-1 viability reduction/autophagy stimulation and in vivo tumor growth down-regulation.	[110]

## Abbreviations

SELEX	Systematic Evolution of Ligands by Exponential Enrichment
ssDNA	single–stranded DNA
AML	acute myeloid leukemia
ALL	acute lymphoblastic leukemia
MM	multiple myeloma
CML	chronic myeloid leukemia
CLL	Chronic Lymphocytic Leukemia
Kd	dissociation constant
Dox	doxorubicin
MTX	methotrexate
OFA/iLRP	Oncofetal antigen/immature laminin receptor protein
PTK7	protein tyrosine kinase 7
NPs	nanoparticles
FRET	fluorescence resonance energy transfer
MRD	Minimal residual disease
RCA	rolling cycle amplification
TPA	two-photon absorption
QCM	quartz crystal microbalance
AuNP	gold nanoparticle
PEC	photoelectrochemical
BP NS	black phosphorus nanosheets
Dau	Daunorubicin
PA	polyvalent aptamers
SWNTs	Single-walled carbon nanotubes
VCR	vincristine
TKI	tyrosine kinase inhibitors
HL	Hodgkin’s lymphoma
NHL	non-Hodgkin’s lymphoma
ALCL	anaplastic large cell lymphoma
ALK	anaplastic lymphoma kinase
BAFF-R	B-cell activating factor receptor
SSC	spliceosome complex
LIGS	LIgand-Guided-Selection
NK	natural killer
PC	plasma cells
BCMA	B-cell maturation antigen
CAM-DR	Cell adhesion-mediated drug resistance
